# Species‐specific regulation of herbivory‐induced defoliation tolerance is associated with jasmonate inducibility

**DOI:** 10.1002/ece3.2953

**Published:** 2017-04-17

**Authors:** Ricardo A. R. Machado, Wenwu Zhou, Abigail P. Ferrieri, Carla C.M. Arce, Ian T. Baldwin, Shuqing Xu, Matthias Erb

**Affiliations:** ^1^ Root‐Herbivore Interactions GroupMax Planck Institute for Chemical EcologyJenaGermany; ^2^ Department of Molecular EcologyMax Planck Institute for Chemical EcologyJenaGermany; ^3^ Institute of Plant SciencesUniversity of BernBernSwitzerland; ^4^ Departamento de EntomologiaUniversidade Federal de ViçosaViçosa (MG)Brazil

**Keywords:** herbivory‐induced tolerance, *Manduca sexta*, regrowth suppression, root carbohydrates

## Abstract

Induced changes in root carbohydrate pools are commonly assumed to determine plant defoliation tolerance to herbivores. However, the regulation and species specificity of these two traits are not well understood. We determined herbivory‐induced changes in root carbohydrates and defoliation tolerance in seven different solanaceous plant species and correlated the induced changes in root carbohydrates and defoliation tolerance with jasmonate inducibility. Across species, we observed strong species‐specific variation for all measured traits. Closer inspection revealed that the different species fell into two distinct groups: Species with a strong induced jasmonic acid (JA) burst suffered from a reduction in root carbohydrate pools and reduced defoliation tolerance, while species with a weak induced JA burst maintained root carbohydrate pools and tolerated defoliation. Induced JA levels predicted carbohydrate and regrowth responses better than jasmonoyl‐L‐isoleucine (JA‐Ile) levels. Our study shows that induced JA signaling, root carbohydrate responses, and defoliation tolerance are closely linked, but highly species specific, even among closely related species. We propose that defoliation tolerance may evolve rapidly via changes in the plant's defense signaling network.

## 1 Introduction

Insect herbivores threaten the performance and fitness of plants. To minimize the impact of herbivory, plants have evolved several mitigating strategies, including induced resistance and tolerance. Induced resistance traits include the production of secondary metabolites and proteins that deter or intoxicate herbivores, or act as cues which increase herbivore predation by natural enemies (Baldwin, 1998; Green & Ryan, 1972; Heil, 2015; Schuman, Barthel, & Baldwin, 2012). However, in the face of adapted herbivore specialists (Berenbaum & Zangerl, 1994; Kaplan & Thaler, 2010; Lindigkeit et al., 1997; Mao, Rupasinghe, Zangerl, Schuler, & Berenbaum, 2006; Zangerl & Berenbaum, 1990) or during insect outbreak and vertebrate herbivory (Crawley, 1989, 1990; Gibson, Brown, & Jepsen, 1987; Hulme, 1994; Machado, McClure, Hervé, Baldwin, & Erb, 2016), defensive traits might be of limited value (Bustos‐Segura, Fornoni, & Núñez‐Farfán, 2014). It is under these circumstances that tolerance might become of central importance for plants as they allow them to compensate for the negative effects of extensive herbivore damage (Agrawal & Fishbein, 2008; Machado et al., 2013; van der Meijden, Wijn, & Verkaar, 1988; Richards & Caldwell, 1985; White, 1973).

In recent years, the increased allocation of photoassimilates toward roots and stems has been proposed to be an induced tolerance mechanism that enables plants to “bunker” carbohydrates and use them for future regrowth (Babst, Ferrieri, Thorpe, & Orians, 2008; Babst et al., 2005; Briske, Boutton, & Wang, 1996; Dyer et al., 1991; Gómez et al., 2012; Holland, Cheng, & Crossley, 1996; Kaplan & Thaler, 2010; Schwachtje et al., 2006). However, increased allocation of photoassimilates to roots and/or stems does not necessarily increase plant tolerance to aboveground herbivores, as the newly allocated carbon may also be used for other purposes, including the synthesis of plant defenses, root respiration, and/or exudation (Barber & Martin, 1976; Clayton et al., 2010; Erb, Lenk, Degenhardt, & Turlings, 2009; Ferrieri, Agtuca, Appel, Ferrieri, & Schultz, 2013; Ferrieri et al., 2005; Frost & Hunter, 2008; Holland et al., 1996; Keith, Oades, & Martin, 1986; Machado et al., 2013; Machado, et al., 2016; Shoji, Yamada, & Hashimoto, 2000). Furthermore, the newly allocated carbon may be sequestered and may not be available for subsequent reallocation to the shoots (Marshall & Sagar, 1965). Interestingly, despite the large number of tracer studies demonstrating a higher net carbon flux to the roots in response to herbivore attack, there is little evidence for an increase in soluble, nonstructural carbohydrates in the roots (Castrillón‐Arbeláez, Martínez‐Gallardo, Arnaut, Tiessen, & Délano‐Frier, 2012; Gómez et al., 2012; Machado et al., 2013; Schwachtje et al., 2006; Steinbrenner, Gómez, Osorio, Fernie, & Orians, 2011). On the contrary, roots are depleted of sugars and starch following leaf–herbivore attack (Gómez et al., 2012; Machado et al., 2013). Therefore, the link between the herbivory‐induced carbon allocation to roots and tolerance remains controversial.

Two recent studies have investigated the link between herbivory‐induced changes in root soluble carbohydrate pools and defoliation tolerance. One study found that aboveground herbivory depletes root carbohydrate pools and constrains the regrowth capacity of *Manduca sexta*‐attacked *Nicotiana attenuata* plants (Machado et al., 2013). Both effects were absent in jasmonate‐deficient transgenic plants impaired in the production of plant resistance metabolites, indicating that jasmonate signaling regulates both resistance and tolerance traits and that they might be subject to trade‐offs (Machado et al., 2013). In contrast, another study found that *Solanum lycopersicum* plants regrew better when attacked by *M. sexta* larvae, despite the herbivore‐induced reduction in root carbohydrates in this species (Gómez et al., 2012; Korpita, Gomez, & Orians, 2014). The contrasting results of these two studies suggest that the connection between herbivory‐induced changes in root carbohydrates and tolerance may differ even between species belonging to the same family.

In this study, we exploited natural variation in plant responses to herbivore attack across the Solanaceae to understand the regulation and species specificity of herbivory‐induced tolerance in this plant family (Korpita et al., 2014; Machado et al., 2013; Xu, Zhou, Pottinger, & Baldwin, 2015; Zavala & Baldwin, 2006). To this end, we evaluated changes in leaf jasmonates, root carbohydrate pools, and regrowth capacity in response to *Manduca sexta* attack in seven solanaceous plant species. *Manduca sexta* is one of the most damaging specialist herbivores of this plant family and can completely defoliate plants (Campbell & Kessler, 2013; Kessler, Halitschke, & Baldwin, 2004; Yamamoto & Fraenkel, 1960). The obtained results provide insights into the regulation of induced defoliation tolerance and its variability across closely related plant species.

## 2 Materials and Methods

### 2.1 Plant material

The following plant species were used in this study: *Petunia axillaris axillaris, Solanum peruvianum* LA2744, *Solanum lycopersicum* LA2696*, Nicotiana miersii, Nicotiana pauciflora, Nicotiana attenuata,* and *Nicotiana obtusifolia*. These species were selected to represent four major Solanaceae clades and cover both intra‐ and intergenus variation. *Nicotiana attenuata* seeds were originally collected in Utah (USA). *Nicotiana miersii*,* N. pauciflora*, and *N. obtusifolia* seeds were obtained from the United States *Nicotiana* germplasm collection. Seeds were multiplied through selfing in the glasshouse. Seeds of *S. lycopersicum* LA2696 and *S. peruvianum* LA2744 were initially obtained from the tomato genetics resource center (TGRC) at Davis University in California (USA) and propagated by bulk pollination. *Petunia axillaris* seeds were derived from a wild accession by self‐fertilization.

### 2.2 Germination and planting conditions

**Figure 1:**
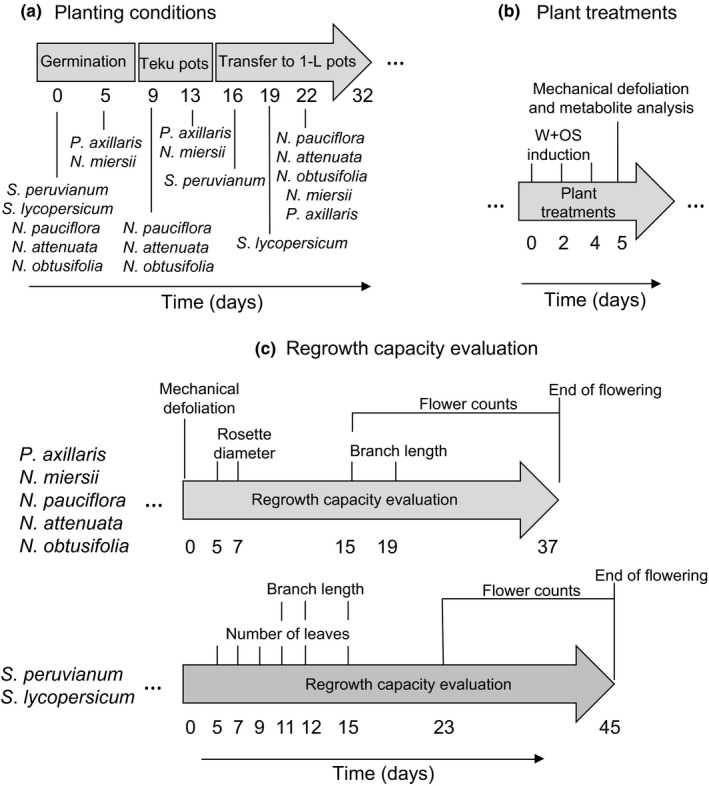
*Manduca sexta*‐induced changes in regrowth capacity evaluation. Planting conditions (a), plant treatments (b), and timing of regrowth capacity evaluation (c)

*Nicotiana attenuata, N. miersii, N. pauciflora, N. obtusifolia,* and *P. axillaris* seeds were germinated on Gamborg's B5 medium as described (Krügel, Lim, Gase, Halitschke, & Baldwin, 2002) (Figure 1). *Nicotiana attenuata* seeds were smoke‐treated to trigger germination. Approximately 9–10 days later, the seedlings were transferred to Teku pots (Pöppelmann GmbH & Co. KG, Lohne, Germany) filled with chunky sand (Raiffeisen GmbH, Germany) for 12 days before transferring them into 1‐L pots filled with sand. *Solanum lycopersicum* LA2696 and *S. peruvianum* LA2744 seeds were germinated directly in Teku pots. Between 16 and 20 days later, the seedlings were transferred into 1‐L pots filled with sand (Figure 1a). This germination procedure was carried out to synchronize the first day of flowering of all plant species. All plants were grown at 45–55% relative humidity and 23–25°C during days and 19–23°C during nights under 16 hr of light (6 a.m.–10 p.m.) supplied by Master Sun‐T PIA Agro 400 or Master Sun‐T PIA Plus 600 W Na lights (Philips, Turnhout, Belgium). Plants were watered twice every day by a flood irrigation system and were fertilized daily with 0.5 g/L Ferty B1 and 1.0 g/L Ca(NO_3_)_2_ × 4 H_2_O (Planta Düngemittel, Regenstauf, Germany).

### 2.3 *Manduca sexta*‐induced changes in regrowth

Upon *M. sexta* attack, *N. attenuata* plants regrow less while *S. lycopersicum* plants regrow better (Korpita et al., 2014; Machado et al., 2013). We determined whether the *M. sexta*‐induced changes in regrowth differ between different species that are grown under similar conditions. All plants were treated during the vegetative stage (Figure 1b). Simulated *M. sexta* attack and the regrowth capacity evaluation were carried out as described (Ferrieri et al., 2015; Machado et al., 2013) (Figure 1b,c). Briefly, a pattern wheel was rolled over the leaf 3–4 times on each side of the midvein and the resulting wounds (W) were immediately treated with 20–30 μl of a 1:5 (v/v) MilliQ water‐diluted *M. sexta* oral secretion (OS) solution. Three to four leaves per plant were treated every time and the treatments were repeated every other day three times to obtain a total of 9–12 treated leaves per plant over 6 days of treatments (Figure 1c). The number of treated leaves and the amount of applied OS to the wounds were kept proportional to the size of the different plant species to standardize induction intensities across plant species and to achieve 30% of the leaves treated on average. Intact plants served as controls (*N* = 19). Simulated herbivory in *N. attenuata* changes regrowth capacity in a similar manner as real *M. sexta* attack (Machado et al., 2013). We used this approach to avoid a bias which may be caused by different feeding intensities of *M. sexta* on the different species (Boer & Hanson, 1984; Yamamoto & Fraenkel, 1960). All plant species evaluated in this study host *M. sexta* under natural conditions (Boer & Hanson, 1984; Campbell & Kessler, 2013; Machado, McClure, Hervé, Baldwin, & Erb, 2016; Yamamoto & Fraenkel, 1960). To specifically evaluate the contribution of belowground tissues to the regrowth capacity of aboveground tissues, all plant species were defoliated 24 h after the last treatment, leaving only the roots and the lowest part of the main stem (0.5–10 cm above the shoot–root junction). Heavy defoliation is commonly observed under natural conditions (Machado, McClure, Hervé, Baldwin, & Erb, 2016). Regrowth was monitored for all species until senescence (Figure 1c) as follows: the number of regrowing leaves was counted, the average rosette diameter was measured, and the cumulative branch length (the sum of the length of all branches) and the number of flowers were quantified. According to their change in regrowth following simulated *M. sexta* attack compared to control plants, plants were classified as tolerant or nontolerant. Tolerant species are those whose regrowth capacity was not affected by simulated *M. sexta* attack. A subset of plants was used for the quantification of root carbohydrates.

### 2.4 *Manduca sexta*‐induced changes in root carbohydrates

To link the observed regrowth patterns to root carbohydrate pools, we measured root carbohydrates upon simulated *M. sexta* herbivory in the same seven species. Root carbohydrate pools (glucose, fructose, sucrose, and starch) were determined by an enzymatic/spectrophotometric method (*N* = 5) (Machado, Arce, Ferrieri, Baldwin, & Erb, 2015; Machado et al., 2013; Smith & Zeeman, 2006; Velterop & Vos, 2001). The sum of glucose, fructose, sucrose, and starch was used as a proxy of total nonstructural carbohydrates (Bokhari, 1977; Cook, 1966; Smith, 1973; White, 1973).

### 2.5 *Manduca sexta*‐induced jasmonate accumulation

Jasmonates regulate induced defoliation tolerance in *N. attenuata* (Machado et al., 2013). To link defoliation tolerance, root carbohydrate pools, and induced jasmonate levels, we measured jasmonate levels upon simulated *M. sexta* attack. Induced jasmonate levels of *N. obtusifolia, N. miersii, N. attenuata,* and *N. pauciflora* were reported in our previous study (Xu et al., 2015). To complete this dataset, we measured jasmonic acid (JA) and jasmonoyl‐L‐isoleucine (JA‐Ile) levels in *P. axillaris*,* S. lycopersicum,* and *S. peruvianum*. Jasmonate measurements across all the plant species were carried out following standard procedures as described (Anssour & Baldwin, 2010; Lou & Baldwin, 2003; Machado et al., 2013; Pearse, Krügel, & Baldwin, 2006; Xu et al., 2015). Briefly, thirty days after germination, one fully expanded leaf was mechanically damaged with a pattern wheel. The resulting wounds were immediately treated with either 20 μl of water (wounding + water; W + W) or 20 μl of 1:5 diluted *M. sexta* oral secretions (wounding + oral secretions; W + OS). Samples were harvested 1 and 2 hr after elicitation, flash‐frozen in liquid nitrogen, and stored at −80°C. Control plants were left intact (*n* = 4). For phytohormone analysis, 200 mg leaf powder was homogenized in 2 ml ethyl acetate containing 10 ng/ml D6‐JA as internal standards. Homogenates were centrifuged at 11,904 g for 30 min at 4°C. Supernatants were collected and evaporated to dryness. Pellets were then reconstituted in 2 ml of 70% (v/v) methanol/water. JA and JA‐Ile levels were measured using liquid chromatography and mass spectrometry (BRUKER DALTONIK, Bremen, Germany) as described (Schäfer, Brütting, Baldwin, & Kallenbach, 2016).

### 2.6 Correlation among *M. sexta*‐induced changes in JA levels, root carbohydrates, and regrowth capacity

To link the different *M. sexta*‐induced phenotypes, we first calculated the magnitude of the *M. sexta*‐induced changes in root carbohydrate pools as the ratio between the total amount of nonstructural root carbohydrates (sum of glucose, fructose, sucrose, and starch) in *M. sexta*‐attacked plants and the amount in nonattacked plants before defoliation. Second, we calculated the magnitude of the *M. sexta*‐induced changes in flower production of the regrowing plants as a measure of induced defoliation tolerance by determining the ratio between the cumulative amount of flowers produced by induced regrowing plants and noninduced regrowing plants. Third, we classified the plant species into low (<0.7 μg/g FW) or high (>1.3 μg/g FW) JA inducers.

### 2.7 Statistics

Unless otherwise stated, statistical tests were carried out with Sigma Plot 12.0 (Systat Software Inc., San Jose, CA, USA) using analysis of variance (ANOVA). Levene's and Shapiro–Wilk tests were applied to determine error variance and normality. Holm–Sidak *post hoc* tests were used for multiple comparisons. The effect of simulated herbivory on all vegetative and reproductive parameters measured in regrowing plants was tested by two‐way repeated‐measures ANOVA with time and treatment as factors. The effect of simulated herbivory on soluble sugars, starch, and total nonstructural carbohydrates was evaluated by two‐way ANOVA with plant species and treatment as factors. Datasets from experiments that did not fulfill the assumptions for ANOVA were natural log‐, root square‐, or rank‐transformed before analysis. To account for the relatedness of the different species in the correlations, the phylogenetic relationship among the studied species was determined based on the internal transcribed spacer region of nuclear 5.8S ribosomal DNA (ITS1 and ITS2) and a chloroplastic—tRNA‐Leu gene, intergene spacer, and the tRNA‐Phe gene (trnLF). The DNA sequences were obtained from GenBank (www.ncbi.nlm.nih.gov/genbank), and the accession numbers are listed in Table S1. Both approaches yielded similar results, and we therefore used the results from ITS1 and ITS2 only (Chase et al., 2003; Goldberg et al., 2010). DNA sequences of ITS were aligned using MUSCLE (v. 3.8.31) and manually curated (Edgar, 2004). A phylogenetic tree based on the DNA sequences was constructed using PhyML (v. 20140206) with a molecular evolution model estimated by jModeltest2 (v.2.1.5) (Darriba, Taboada, Doallo, & Posada, 2012; Guindon et al., 2010). Using the constructed phylogenetic tree (Figure S1), we estimated the relationship between herbivory‐induced regrowth capacity, root carbohydrates, and jasmonates by phylogenetic generalized least squares (PGLS) models in R (v.3.1.1) using the package caper (Orme et al., 2012; R Development Core Team 2012).

## 3 Results

### 3.1 The regrowth response upon simulated *M. sexta* attack is species specific

**Figure 2:**
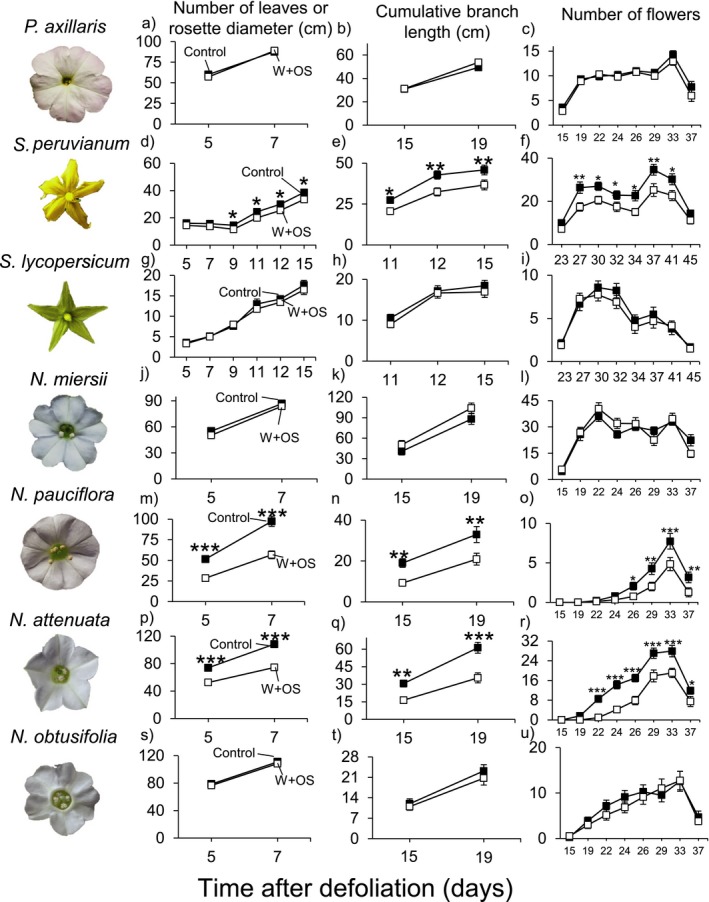
Impact of *Manduca sexta* attack on regrowth capacity of seven solanaceous plant species. Average (±*SE*) rosette diameter (a, m, p, s), number of leaves (d, g, j), cumulative branch length (b, e, h, k, n, q, t), and number of flowers (c, f, i, l, o, r, u) of regrowing *Petunia axillaris* (a–c), *Solanum peruvianum* (d–f), *Solanum lycopersicum* (g–i), *Nicotiana miersii* (j–l), *Nicotiana pauciflora* (m–o), *Nicotiana attenuata* (p–r), and *Nicotiana obtusifolia* (s–u) plants. Asterisks indicate significant differences (*:*p *<* *.05; **: *p *<* *.01; ***: *p *<* *.001) between W + OS‐treated (*n* = 19) and control plants (*n* = 19) within each plant species and time point. Control (closed squares): intact plants; W + OS (open squares): plants treated with wounding and *M. sexta* oral secretions (simulated *M. sexta* attack)

Across the seven tested solanaceous plant species, we observed neutral to negative regrowth patterns in plants under simulated *M. sexta* attack compared to nonelicited controls (Figure 2). While simulated *M. sexta* attack did not affect either the vegetative growth or reproductive output of *P. axillaris*,* S. lycopersicum, N. miersii,* and *N. obtusifolia*, it reduced the leaf number, branch length, and number of flowers in *S. peruvianum*,* N. pauciflora,* and *N. attenuata* (Figure 2).

### 3.2 The root carbohydrate response upon simulated *M. sexta* attack is species specific

**Figure 3:**
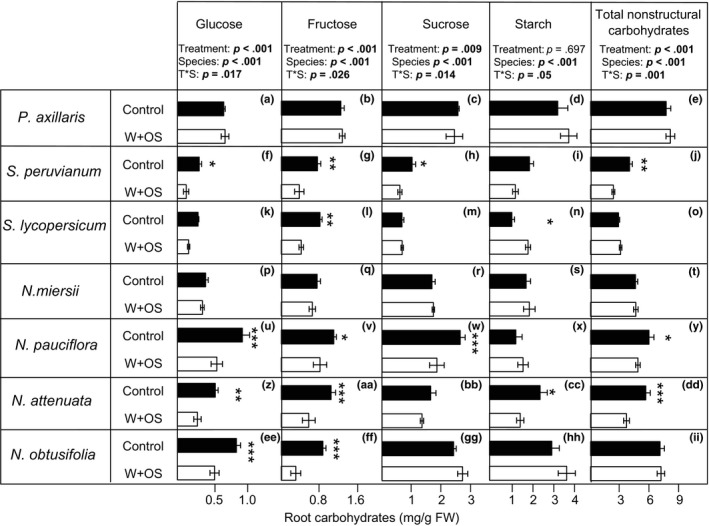
*Impact of M. sexta attack on root carbohydrate pools of seven solanaceous plant species*. Average (±SE) glucose (a, f, k, p, u, z, ee), fructose (b, g, l, q, v, aa, ff), sucrose (c, h, m, r, w, bb, gg), starch (d, i, n, s, x, cc, hh) and total nonstructural carbohydrates (e, j, o, t, y, dd, ii) of *Petunia axillaris* (a‐e), *Solanum peruvianum* (f‐j), *Solanum lycopersicum* (k‐o), *Nicotiana miersii* (p‐t), *Nicotiana pauciflora* (u‐y), *Nicotiana attenuata* (z‐dd) and *Nicotiana obtusifolia* (ee‐ii) plants. Asterisks indicate significant differences (*: *P*<0.05; **: *P*<0.01; ***: *P*<0.001) between W+OS‐treated (*n*=5) and control plants (*n*=5) within each plant species and metabolite. Control: intact plants; W+OS: plants treated with wounding and *M. sexta* oral secretions (simulated *M. sexta* attack).

Similar to the measured regrowth responses, we found neutral to negative effects of simulated *M. sexta* herbivory on root carbohydrate pools (Figure 3). Total soluble, nonstructural carbohydrate pool—calculated as the total amount of glucose, fructose, sucrose, and starch—in *P. axillaris*,* S. lycopersicum, N. miersii,* and *N. obtusifolia* plants remained unaffected by leaf induction (Figure 3). By contrast, a significant reduction in total root carbohydrates was observed in *S. peruvianum*,* N. pauciflora,* and *N. attenuata*. One exception was *N. obtusifolia*, in which simulated *M. sexta* herbivory reduced root glucose and fructose levels, but not total nonstructural carbohydrates due to a slight, nonsignificant increase in starch levels. The only positive response was observed for starch levels in *S. lycopersicum*, which increased in the roots of leaf‐induced plants. Total soluble carbohydrates were not changed in the roots of *S. lycopersicum*.

### 3.3 Jasmonate induction upon simulated *M. sexta* attack varies greatly across plant species

Consistent with our previous studies, high variation in *M. sexta*‐induced JA levels were observed across species (Anssour & Baldwin, 2010; Lou & Baldwin, 2003; Machado et al., 2013; Pearse et al., 2006). Across species, we observed that simulated herbivory attack induces a rapid accumulation of JA and JA‐Ile in the leaves, with concentrations peaking after 1 hr of elicitation. In *N. obtusifolia*,* P. axillaris*,* S. lycopersicum,* and *N. miersii,* simulated herbivory induced JA levels below 0.7 μg/g FW (Figure S2, Table S2), while JA concentrations in *N. pauciflora*,* N. attenuata,* and *S. peruvianum* reached concentrations above 1.3 μg/g FW (Figure S2, Table S2). JA and JA‐Ile levels positively, but weakly, correlated across species (Figure S3).

### 3.4 *Manduca sexta*‐induced changes in JA levels and root carbohydrates are correlated across species

**Figure 4:**
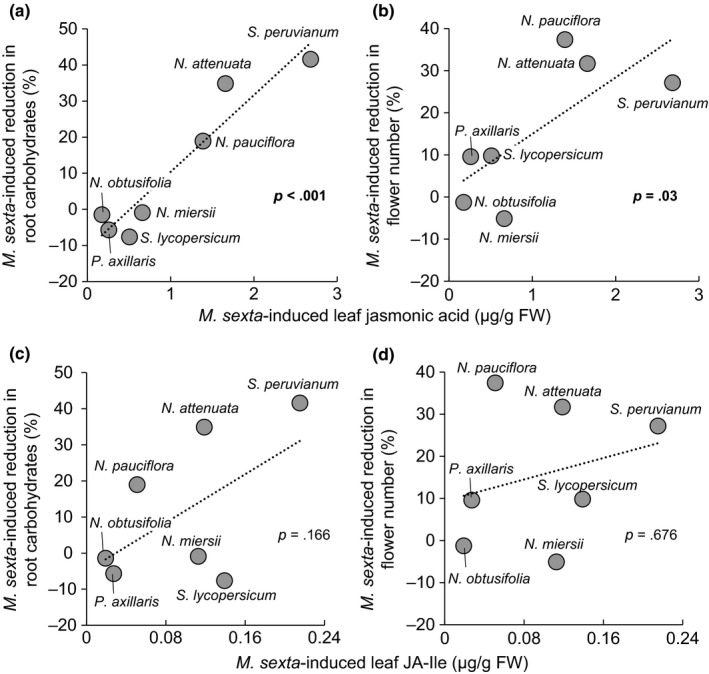
*Manduca sexta*‐induced changes in jasmonic acid (JA), but not jasmonoyl‐L‐isoleucine, are positively correlated with regrowth capacity and root carbohydrate pools. Correlation between *M. sexta*‐induced leaf JA and the magnitude of the *M. sexta*‐induced root carbohydrate depletion at the beginning of the regrowth phase (a) and the magnitude of *M. sexta*‐induced reduction of flower production by regrowing plants (b). Correlation between the *M. sexta*‐induced leaf JA‐Ile and the magnitude of the *M. sexta*‐induced root carbohydrate depletion at the beginning of the regrowth phase (c) and the magnitude of *M. sexta*‐induced reduction of flower production by regrowing plants (d). Correlations were tested using phylogenetic generalized least squares (pgls)

**Figure 5:**
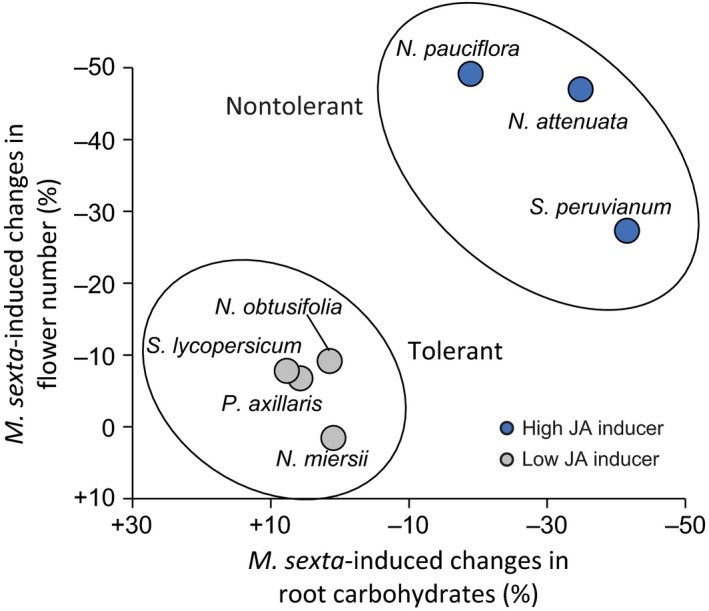
*Manduca sexta*‐induced jasmonic acid (JA) levels, total nonstructural root carbohydrates, and regrowth capacity are strongly correlated across species. Grouping effect in the magnitude of *M. sexta*‐induced root carbohydrate depletion at the beginning of the regrowth phase (*x*‐axis) and the magnitude of *M. sexta*‐induced reduction of flower production by regrowing plants (*xy*‐axis) across plant species. Blue circles designate high (more than 1.3 μg/g FW) and gray circles low (<0.7 μg/g FW) JA inducers

Linear correlations revealed a positive relationship between *M. sexta*‐induced leaf JA levels and the magnitude of *M. sexta*‐induced changes in root carbohydrate pools (*p *<* *.001) and flower production (*p *=* *.03) across species (Figure 4a,b). In contrast, *M. sexta*‐induced leaf JA‐Ile levels did not correlate with root carbohydrate levels (*p *=* *.166) or flower production (*p *=* *.676) (Figure 4c,d). Two‐dimensional component analysis revealed a clear grouping effect that separated nontolerant from tolerant species according to the magnitude of induced JA, root carbohydrate depletion, and regrowth capacity (Figure 5). Plant species that induced high levels of JA in response to simulated *M. sexta* attacked suffered from root carbohydrate depletion and suppression of regrowth, while species that induced low levels of JA did not suffer from carbohydrate depletion or a reduction in flower production (Figure 5).

## 4 Discussion

In this study, we show that herbivory‐induced changes in leaf JA, root carbohydrates, and defoliation tolerance are strongly correlated, but highly variable between species. Our findings are consistent with the hypothesis that induced changes in root carbohydrate pools are controlled by jasmonates and are important determinants of plant tolerance to defoliation.

We observed a strong grouping effect across *M. sexta*‐induced phenotypes and plant species. The first group consists of plant species which, in response to simulated *M. sexta* attack, produce high levels of JA, deplete root carbohydrate reserves, and suffer from a reduced regrowth capacity. The second group includes plant species which produce low levels of JA, maintain root carbohydrate reserves, and maintain their regrowth capacity upon simulated *M. sexta* attack. From a mechanistic point of view, the strong correlation of these three traits across species may be due to several reasons. First, it is likely that the changes in root carbohydrates directly determine a plant's capacity to produce new photosynthetic tissues at the end of the defoliation process. Plants with lower levels of soluble, nonstructural carbohydrates in the roots often regrow smaller shoots (Bokhari, 1977; Lee, Donaghy, Sathish, & Roche, 2009; Machado et al., 2013; Smith & Silva, 1969). Second, both traits may share a common regulatory basis. Variation in both regrowth and root carbohydrate depletion is tightly associated with jasmonate signaling in *N. attenuata* (Machado et al., 2013). In both transgenic and naturally occurring jasmonate‐deficient *N. attenuata* lines, neither carbohydrate depletion nor a reduction in regrowth is observed, a result in stark contrast to those of jasmonate‐competent plants (Machado et al., 2013). Together with the results presented here, these findings strongly suggest that jasmonates regulate defoliation tolerance by altering root carbohydrate pools and that this mechanism is conserved across different species of the Solanaceae.

Across plant species, we observed high variability in the profiled traits, even within the genus *Nicotiana*, which included four closely related *Nicotiana* species. The pattern found here is similar to the pattern of *M. sexta*‐induced defense traits observed in *Solanum* (Haak, Ballenger, & Moyle, 2014). Regulating tolerance through a major stress hormone pathway may have enabled plants to rapidly change their defoliation tolerance in a changing environment as small genetic changes may have been sufficient to alter this phenotype. More detailed phylogenetic studies will be needed to gain a better picture of the evolution of induced defolation tolerance in the Solanaceae.

A phylogenetic study across 36 *Asclepias* species uncovered no clear correlation between resistance traits and regrowth ability (Agrawal & Fishbein, 2008), and evidence is emerging that plants may employ mixed strategies to survive herbivore attack (Núñez‐Farfán, Fornoni, & Valverde, 2007). At first glance, our results may lead to the conclusion that a negative association between resistance and defoliation tolerance would be very likely for the Solanaceae, as jasmonates regulate resistance traits positively (Jimenez‐Aleman, Machado, Baldwin, & Boland, 2017) and defoliation tolerance negatively (Jimenez‐Aleman, Machado, Görls, Baldwin, & Boland, 2015; Machado et al., 2013). However, while resistance factors are in large part controlled by JA‐Ile, we found strong negative correlations between JA, but not JA‐Ile and defoliation tolerance. Could JA predominantly regulate tolerance traits, by, for example, regulating carbon allocation (Machado et al., 2015), while JA‐Ile regulates resistance traits? Several studies suggest specific signaling roles of JA (Li et al., 2005; Machado et al., 2015; Schilmiller, Koo, & Howe, 2007; Wang, Allmann, Wu, & Baldwin, 2008). Investigating whether the diversification of jasmonate signaling has enabled plants to uncouple tolerance and resistance processes is an exciting prospect of this work.

## Conflict of Interest

None declared.

## Data Accessibility

All datasets are provided as supplementary material.

## Author Contribution

RM designed and carried out all the experiments, analyzed data, and wrote the manuscript. WZ designed and carried out experiments and contributed to writing the manuscript. AF and CA carried out experiments and contributed to writing the manuscript. IT analyzed data and contributed to writing the manuscript. ME conceived the study, designed experiments, analyzed data, and wrote the manuscript. SX designed experiments, analyzed data, and wrote the manuscript. All coauthors read and approved the final version of the manuscript.
