# Supplementary material for: Species‐specific regulation of herbivory‐induced defoliation tolerance is associated with jasmonate inducibility
Source: Ecol Evol. 2017 Apr 17;7(11):3703–12. doi: 10.1002/ece3.2953 (PMC5468159; doi:10.1002/ece3.2953)
Supplement: Supplementary file 1 [file ECE3-7-3703-s001.pdf]

Figure S1

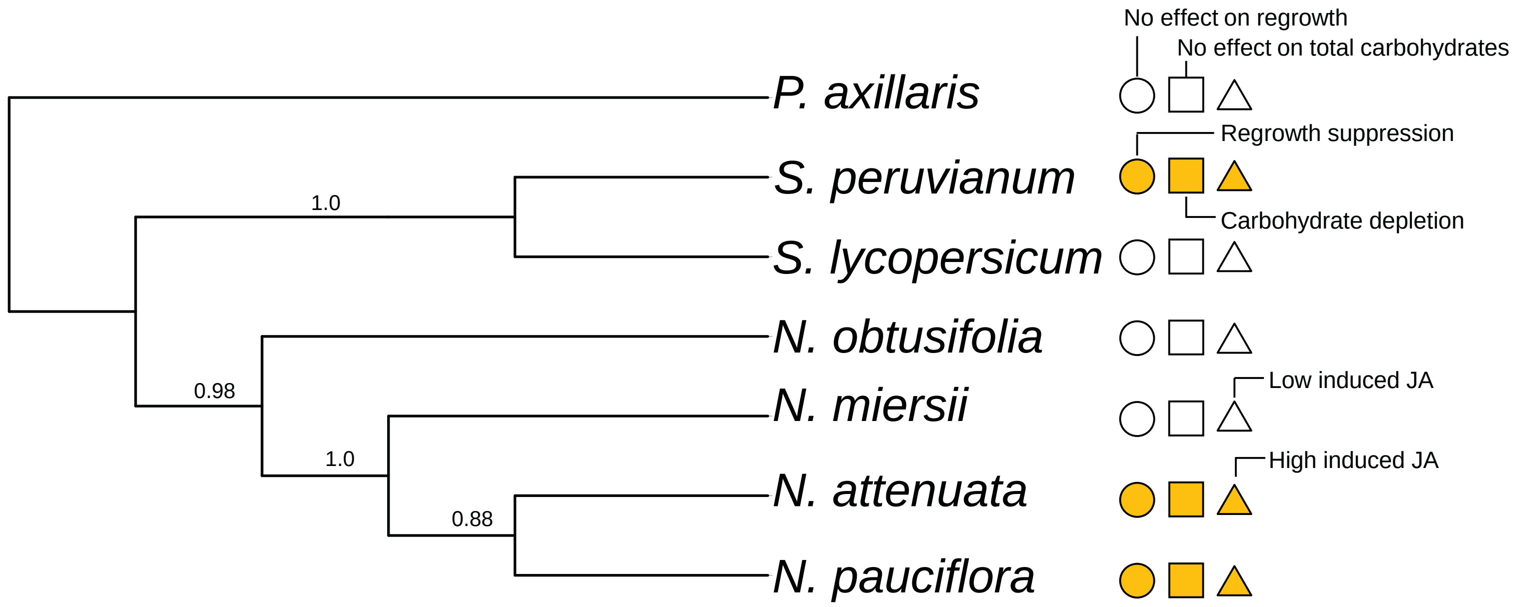
No effect on regrowth
No effect on total carbohydrates
P. axillaris
Regrowth suppression
S. peruvianum
Carbohydrate depletion
1.0
S. lycopersicum
N. obtusifolia
0.98
N. miersii
Low induced JA
1.0
High induced JA
N. attenuata
0.88
N. pauciflora

Figure S2

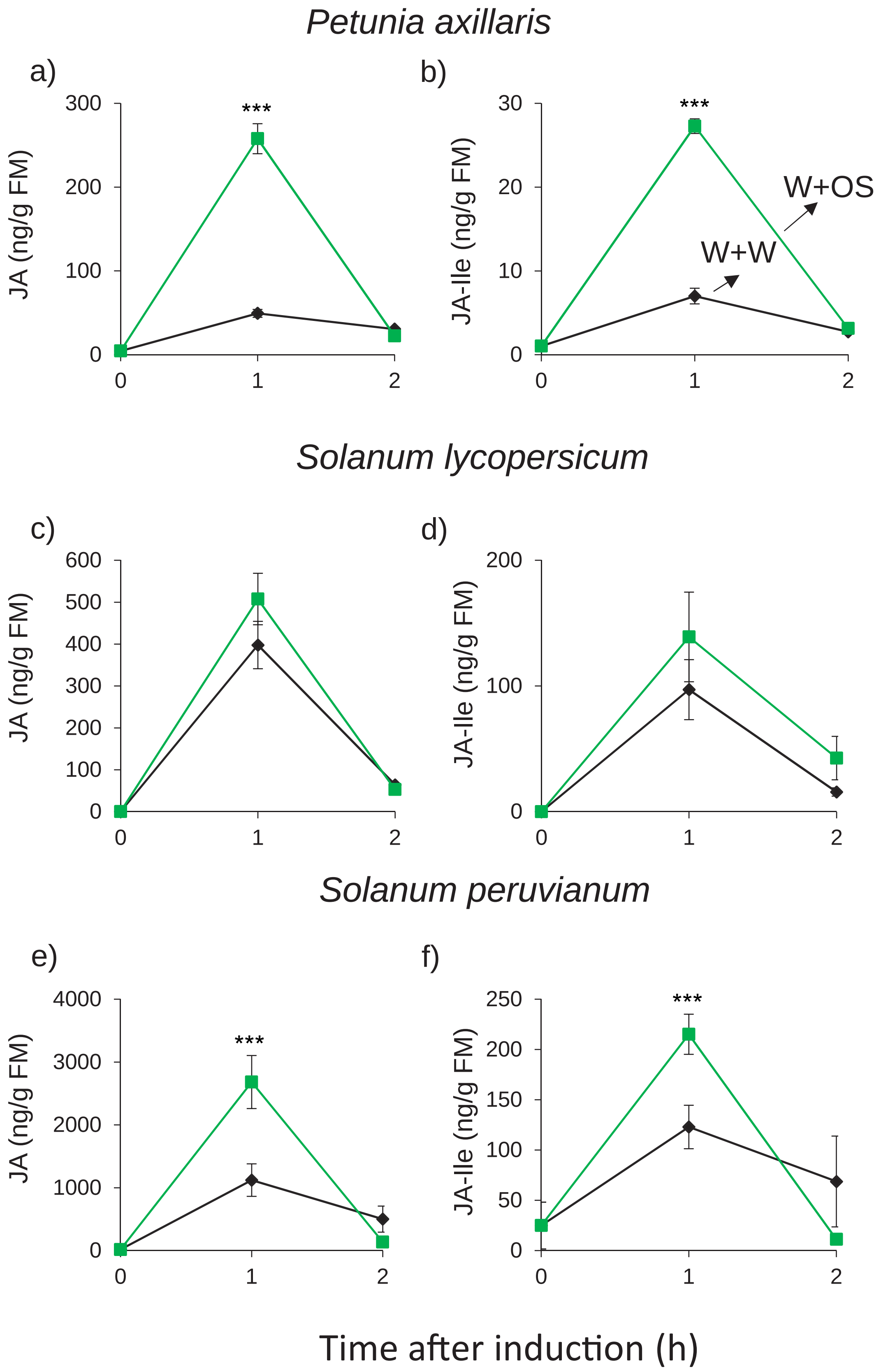

Petunia axillaris
a)
JA (ng/g FM)
300
200
100
0
***
0
1
2
b)
JA-Ile (ng/g FM)
30
20
10
0
***
W+OS
W+W
0
1
2
Solanum lycopersicum
c)
JA (ng/g FM)
600
500
400
300
200
100
0
0
1
2
d)
JA-Ile (ng/g FM)
200
100
0
0
1
2
Solanum peruvianum
e)
JA (ng/g FM)
4000
3000
2000
1000
0
***
0
1
2
f)
JA-Ile (ng/g FM)
250
200
150
100
50
0
***
0
1
2
Time after induction (h)

Figure S3

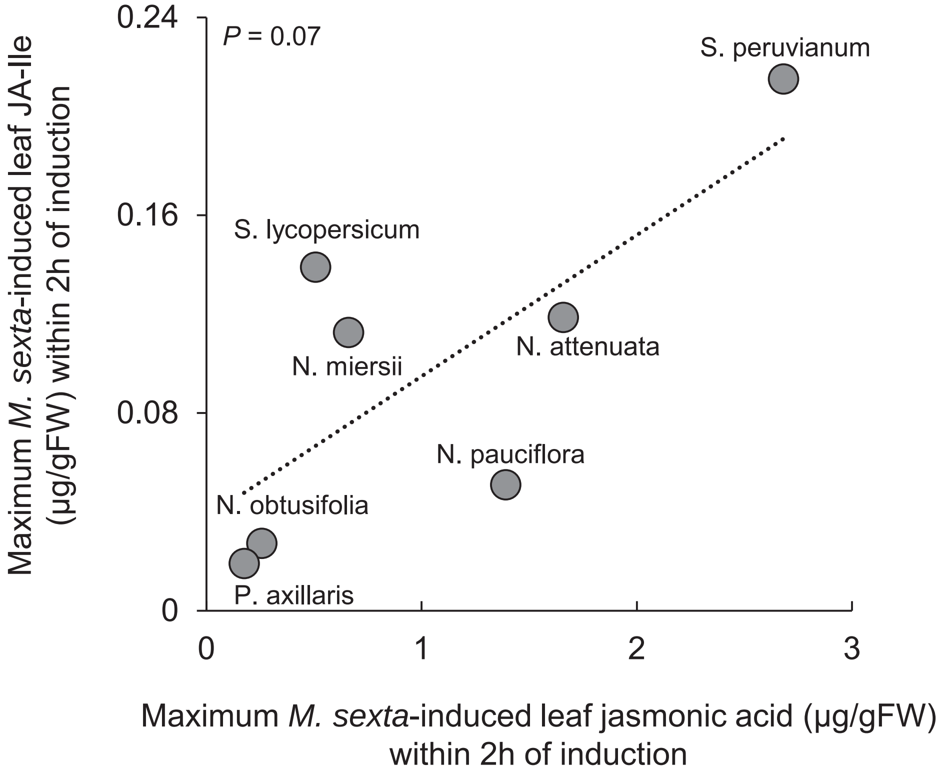

Maximum M. sexta-induced leaf JA-Ile (µg/gFW) within 2h of induction
0.24
0.16
0.08
0
P = 0.07
S. peruvianum
S. lycopersicum
N. miersii
N. attenuata
N. pauciflora
N. obtusifolia
P. axillaris
0
1
2
3
Maximum M. sexta-induced leaf jasmonic acid (µg/gFW) within 2h of induction

Table S1. Gene bank accession numbers of internal transcribed spacer (ITS) regions of nuclear 5.8S ribosomal DNA (ITS1 and 2).

Plant Species	**ITS1 and 2**
N. attenuata	AJ492427.1
N. miersii	AJ492429.1
N. obtusifolia	GQ120451.1
N. pauciflora	AJ492428.1
P. axillaris	AJ300213.1
S. lycopersicum	KC213749.1
S. peruvianum	AJ300210.1

Table S2. Comparison of maximum simulated *M. sexta* attack induced jasmonic acid (JA) and jasmonoyl-L-isoleucine (JA-Ile) levels within 2 h after elicitation across seven solanaceous species.

**Species**	**Maximum induced JA within 2h (ng/gFW)**	**Maximum induced JA-Ile within 2h (ng/gFW)**	**Data from**
N. obtusifolia	175.98 ± 15.96	19.2 ± 3.00	Xu *et al*. 2015
P. axillaris	257.73 ± 17.98	27.28 ± 0.86	This study
S. lycopersicum	507.58 ± 61.53	139.01 ± 35.63	This study
N. miersii	661.61 ± 81.33	112.61 ± 24.22	Xu *et al*. 2015
N. pauciflora	1390.80 ± 162.99	50.98 ± 3.75	Xu *et al*. 2015
N. attenuata	1659.55 ± 158.66	118.68 ± 5.55	Xu *et al*. 2015
S. peruvianum	2681.30 ± 422.37	215.11 ± 19.94	This study
